# A novel approach to differentiate rat embryonic stem cells *in vitro* reveals a role for RNF12 in activation of X chromosome inactivation

**DOI:** 10.1038/s41598-019-42246-2

**Published:** 2019-04-15

**Authors:** Aristea Magaraki, Agnese Loda, Cristina Gontan, Sarra Merzouk, Esther Sleddens-Linkels, Stephen Meek, Willy M. Baarends, Tom Burdon, Joost Gribnau

**Affiliations:** 1 Erasmus University Medical Center, Department of Developmental Biology, Wytemaweg 80, 3015 CN Rotterdam, The Netherlands; 2 Present Address: European Molecular Biology Laboratory (EMBL), Meyerhof Str. 1, 69117 Heidelberg, Germany; 3 The Roslin Institute and R(D)VS, University of Edinburgh, Easter Bush, Midlothian, EH25 9RG Scotland

## Abstract

X chromosome inactivation (XCI) is a mammalian specific, developmentally regulated process relying on several mechanisms including antisense transcription, non-coding RNA-mediated silencing, and recruitment of chromatin remodeling complexes. *In vitro* modeling of XCI, through differentiation of embryonic stem cells (ESCs), provides a powerful tool to study the dynamics of XCI, overcoming the need for embryos, and facilitating genetic modification of key regulatory players. However, to date, robust initiation of XCI *in vitro* has been mostly limited to mouse pluripotent stem cells. Here, we adapted existing protocols to establish a novel monolayer differentiation protocol for rat ESCs to study XCI. We show that differentiating rat ESCs properly downregulate pluripotency factor genes, and present female specific *Xist* RNA accumulation and silencing of X-linked genes. We also demonstrate that RNF12 seems to be an important player in regulation of initiation of XCI in rat, acting as an *Xist* activator. Our work provides the basis to investigate the mechanisms directing the XCI process in a model organism different from the mouse.

## Introduction

In mammals, X chromosome inactivation (XCI) ensures dosage compensation of sex chromosomal genes between females (XX) and males (XY)^1,2^. The process of XCI occurs early during female embryonic development and is mediated by a multitude of epigenetic mechanisms that result in the complete transcriptional inactivation of one entire X chromosome within the nucleus of every female somatic cell. In eutherians, initiation of XCI is mediated by the long non-coding RNA *Xist*^3–6^. During XCI, *Xist* RNA spreads *in cis* along the entire length of the X chromosome and triggers chromosome-wide silencing of X-linked genes by recruitment of a plethora of chromatin remodelers^7–10^. The study of XCI relies both on *in vivo* and *in vitro* models that allow genetic manipulation of the factors involved, and the vast majority of our current knowledge has been achieved by using the mouse as a model organism. *In vivo* studies have shown that XCI starts around the 4–8 cell stage of female mouse embryonic development and is initially imprinted (iXCI), resulting in exclusive inactivation of the paternal X chromosome (Xp)^11–14^. Later in development, at the blastocyst stage (~E4.5), the Xp becomes reactivated in the inner cell mass (ICM) of the embryo, whereas iXCI persists in the extra-embryonic lineages^12,13^. Reactivation of Xp in the ICM is then followed by random inactivation (rXCI) of either the paternal or maternal X chromosome in cells of the developing epiblast. *In vitro*, mouse embryonic stem cells (mESCs) have been extensively used to model rXCI. In fact, undifferentiated mESCs carry two active X chromosomes and faithfully mimic the pluripotent environment of the ICM, whereas their differentiation results in random inactivation of one of the two X chromosomes. Mouse ESC-based *in vitro* studies have led to the discovery of the long non-coding gene *Tsix*, which is transcribed antisense to *Xist* and represents the major repressor of *Xist* up-regulation at the onset of XCI^15–18^. XCI is tightly linked to loss of the pluripotent state^19,20^ and several pluripotency factors including NANOG, SOX2, OCT4, REX1 and PRDM14 have been described to function as XCI-inhibitors either by directly inhibiting *Xist* expression or by enhancing *Tsix*^21–24^. Activation of XCI is mediated by the X-linked E3 ubiquitin ligase RNF12 involved in dose-dependent degradation of REX1^25,26^. Since *Rnf12*^−/−^ mouse ESCs fail to upregulate *Xist* upon differentiation, *Rnf12* has been suggested to be essential for the initiation of XCI^27^. *In vivo*, female embryos that maternally inherited an *Rnf12* null allele fail to initiate iXCI on the paternal X chromosome and die in utero^28^, whereas RNF12 has been shown to be dispensable for rXCI to occur^29^. Thus, further research is needed to better understand the observed discrepancies between *in vivo* and *in vitro* studies. Interestingly, the study of XCI in female pre-implantation embryos from different species suggested that the epigenetic processes that mediate XCI might be more heterogeneous than expected. Indeed, iXCI occurs in the extra-embryonic lineages of rat and cow^30–32^ whereas in other species such as human, monkey, horse, pig and rabbit, rXCI has been exclusively observed in both embryonic and extra-embryonic tissues^33,34^. Comparative analysis of *Xist* RNA expression dynamics and X-linked gene silencing between rabbit and human pre-implantation embryos confirmed substantial diversity in the timing and regulation of XCI initiation among mammals, with cells of the human ICM showing two active X chromosomes regardless of *Xist* RNA coating^30^. In addition, the overall *Xist* gene structure appears to be conserved in all placental mammals^32^_._ For instance, comparative analysis of mouse and rat *Xist* sequence have shown that most exons are conserved between the two species, including repeat A, the 5′ element which is required to achieve proper gene silencing during XCI^35,36^. On the other hand, *Tsix’s* sequence evolved rapidly and differs between species^37–40^. Finally, *Tsix* antisense transcription through the *Xist* promoter is absent in human^41,42^, but appears to be conserved in rodents^43^. Interestingly, differentiation of mouse-rat allodiploid ES cells leads to specific primary inactivation of the mouse X chromosome^44^. This mouse allele-biased expression of *Xist* has been proposed to result from the higher expression of *Tsix* from the rat allele, interfering with expression of *Xist in cis*^44^.

Clearly, the development of novel *in vitro* systems derived from different species is necessary to reach a comprehensive understanding of the XCI process. However, although the induced pluripotent stem cell (iPSC) technology has allowed the generation of several ES cell-like lines from different species^45–47^, except for mouse iPSCs^48^, the characterization of the X chromosome status and the generation of *in vitro* differentiation protocols that recapitulate XCI have proven to be challenging^49–51^. Specific culture conditions that allow studying dosage compensation in human ES cells (hESCs) have also been established^52,53^. These studies provided *in vitro* systems that resemble the pre-XCI state of human pre-implantation embryos, but also showed that hESCs do not undergo random XCI upon differentiation. Thus, these observations suggest the presence of an epigenetic memory that may affect the faithful recapitulation of XCI as it occurs upon early human development, and highlight the need to further optimize cell culture conditions^53^. In this context, rat ES cells (rESCs)^54–62^, represent a powerful alternative to mESC to study the dynamics of XCI. Although closely related, mouse and rat diverged ~12–24 million years ago^63^, and there are significant differences between the two species. The large-scale structure of the rat genome is closer to the human genome than the mouse genome^64^, and the X chromosome has undergone a higher number of rearrangements in the rodent lineages compared to other mammalian orders^65,66^. Furthermore, compared to the mouse, the rat offers several advantages as a model system for modelling human diseases^67,68^. In functional genetic studies, the use of rat as a model system has been limited by the lack of genome engineering tools that allow efficient genetic manipulation of rESC. In this context, the development of novel genomic resources that followed the sequencing of the rat genome^69^ and the establishment of the CRISPR/Cas9 technology for genome editing rapidly enhanced the generation of transgenic rat models^70,71^ providing the basis to perform genetic studies.

Here, we set out to generate a robust *in vitro* system that could faithfully mimic the dynamics of XCI in rat. By developing a neuronal monolayer differentiation protocol for rESCs adapted from Vaskova and colleagues^72^, we were able to follow several aspects of XCI regulation in rat. Similar to mouse, we were able to observe (I) *Xist* up-regulation at an early stage of rESC differentiation followed by (II) transcriptional inactivation of X-linked genes and (III) H3K27me3 accumulation on the inactive X chromosome (Xi). In addition, (IV) overexpression experiments in rESCs confirmed that the REX1-RNF12 axis of *Xist* regulation is most likely conserved between rat and mouse. Thus, our data has established the technical basis to study the dynamics of XCI in a different system from the mouse and suggests that specific aspects of XCI may be conserved in mouse and rat.

## Results

### *In vitro* neuronal differentiation of rESCs

**Figure 1:**
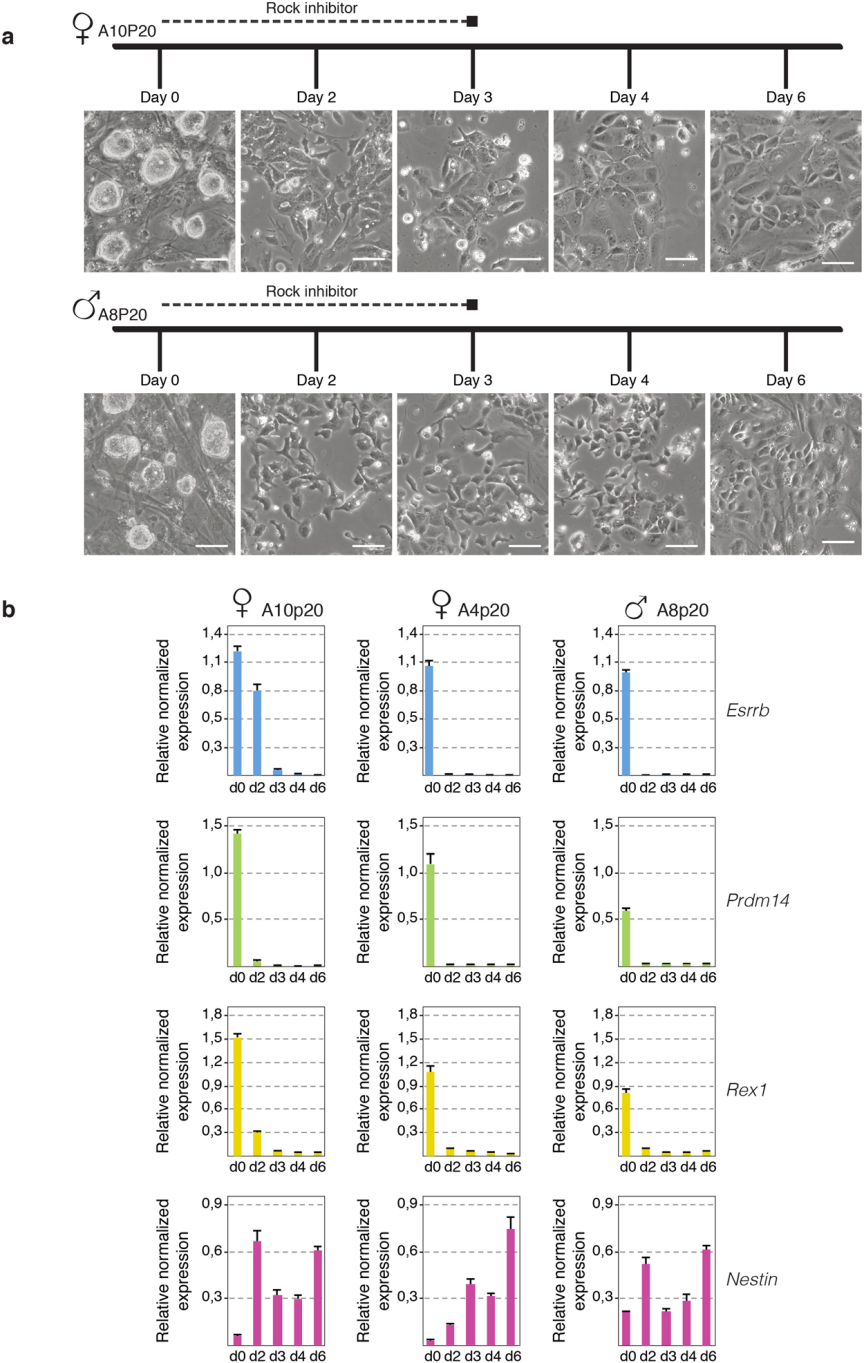
Neuronal differentiation of rESCs. (**a**) Schematic representation of our neuronal differentiation strategy. Brightfield images of female (A10p20, upper panel) and male (A8p20, lower panel) rESCs at day 0 and day 2, 3, 4 and 6 of differentiation are shown. Scale bars represent 80 μm. (**b**) qPCR analysis of *Esrrb*, *Prdm14*, *Rex1* (pluripotency factors) and *Nestin* (neuronal differentiation marker) expression levels normalised to *Actinb* in female (A10p20, A4p20) and male (A8p20) differentiating rESCs. Error bars represent standard deviations of two technical replicates from one experiment.

*In vitro* differentiation of mESCs towards different functional cell types including neurons, cardiomyocytes, hepatocytes and pancreatic cells can be efficiently achieved by several established protocols^73^. Usually, differentiation strategies are based on the formation of embryoid bodies (EB) followed by growth-factor-mediated induction of early progenitor cells to differentiate into their respective lineages. Despite of the growing list of differentiation protocols for mESCs, differentiation of rESCs is extremely difficult to achieve *in vitro*. To date, only two strategies have been described in which rESCs were triggered to differentiate into either cardiomyocytes or neuronal precursors and in these differentiation protocols MEK and GSK3β inhibitors, that are commonly used for ESC culture, are always present in low concentrations in the differentiation media^74,75^. XCI is closely linked to loss of pluripotency, and the presence of an inactive X chromosome provides a powerful readout for cell differentiation. Several rESCs derived from different rat inbred strains were differentiated, including three pure Lewis lines (LEW) (A4p20, A9p20, A10p20), and two lines of a mixed background of dark agouti (DA) and Sprague-Dawley (SD) (135-7, 141-6). All ESC lines displayed dome shaped morphology and expressed the pluripotency factors *Rex1*, *Prdm14*, and *Esrrb* (Fig. 1A,B). We initially set out to assess rat XCI after inducing rESCs differentiation according to protocols, which included MEK and GSK3β inhibitors. Although female cells appeared to be morphologically differentiated into neuronal precursors, we observed enrichment of the H3K27me3 histone modification (hallmark of gene silencing upon XCI) in only 20% of the cell population, (Supplementary Fig. 1A). *Xist* RNA FISH analysis, detecting *Xist* and *Tsix*, revealed absence of *Xist* accumulation, and confirmed the presence of small pinpoint transcription signals on both X chromosomes in most cells, likely representing *Tsix* transcription signals, as observed in mouse (Supplementary Fig. 1B). These results indicated our rESCs to be compromised to initiate XCI. We therefore set out to optimize the protocol in such a way that differentiation would include proper initiation of XCI. The absence of *Xist* accumulation and XCI might be related to the presence of MEK and GSK3β inhibitors, stabilizing the pluripotent state, and potentially resulting in expression of factors that repress *Xist*^20,22–24,57^. We therefore adapted the neuronal differentiation protocol initially described by Peng *et al*. and Vaskova *et al*.^72,75^ as follows: (I) both 2i inhibitors were completely eliminated starting from day 1 of neuronal differentiation, (II) the concentration of ROCK (rho-associated protein kinase) inhibitor, shown to prevent dissociation-induced apoptosis in cultured human ES cells^46,76^, was increased, (III) cells were seeded on laminin, (IV), a greater number of rESCs was used for differentiation and finally (V) differentiation cultures were serum and FGF2 free. Using these modified conditions, we were able to maintain viable differentiating male and female rESCs in the absence of 2i inhibitors (Fig. 1A). Importantly, qPCR analysis of both pluripotency and differentiation marker expression levels at different time points upon differentiation confirmed efficient downregulation of the pluripotency factors *Esrrb*, *Prdm14* and *Rex1*, and parallel up-regulation of the neuronal precursor marker *Nestin* (Fig. 1B).

### Female rESCs undergo XCI upon *in vitro* neuronal differentiation

**Figure 2:**
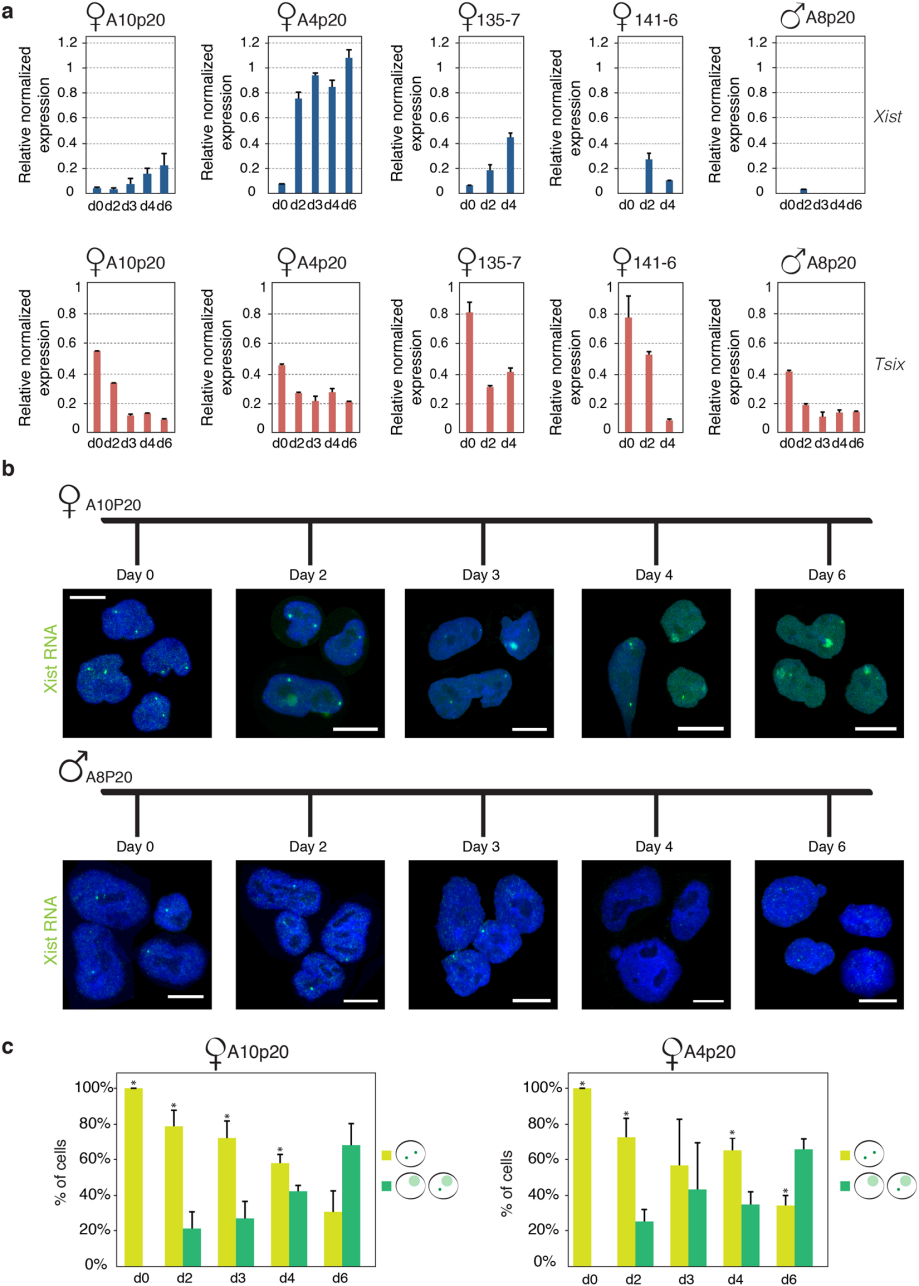
Monoallelic upregulation of *Xist* RNA upon female rESCs differentiation. (**a**) *Xist* and *Tsix* qPCR expression analysis in female (A10p20, A4p20, 135-7, 141-6) and male (A8p20) differentiating rESCs. Expression levels of *Xist* and *Tsix* at different time points upon neuronal differentiation are shown. In the *Xist* panel error bars represent standard deviation of technical replicates from one experiment. In the *Tsix* panel error bars in A10P20, A4P20 and A8P20 represent standard deviations of technical replicates from one experiment and in 135-7 and 141-6 standard deviations of technical replicates from two experiments. (**b**) Representative images of *Xist* RNA FISH (green) analysis upon differentiation of female (A10p20-upper panel) and male (A8p20-lower panel) rESCs. DNA is stained with DAPI (blue). Scale bars represent 10 μm. (**c**) Quantification of relative number of *Xist* RNA signals (pinpoints or clouds) in A10p20 (left graph) and A4p20 (right graph) female rESCs at day 0, 2, 3, 4 and 6 upon neuronal differentiation. *Xist* clouds were never observed in male nuclei. More than 100 nuclei were counted for each time point and for each experiment. Error bars represent standard deviation of two experimental replicates. Asterisks indicate significant difference between the two groups (2 pinpoints versus cloud with or without pinpoint) within each differentiation time point. P-values are provided: p-value_A10-d0_ < 0.0001, p-value_A10-d2_ = 0.02, p-value_A10-d3_ = 0.03, p-value_A10-d4_ = 0.03 and p-value_A4-d0_ < 0.0001, p-value_A4-d2_ = 0.03, p-value_A4-d4_ = 0.05, p-value_A4-d6_ = 0.03.

We then addressed the question of whether differentiating rESCs without the supplement of 2i inhibitors would facilitate XCI. To this end, four independent female rESC lines were differentiated and the *Xist* RNA expression level was assessed by qPCR analysis at different time points upon neuronal differentiation. Importantly, in order to assess the sex-specific regulation of *Xist* RNA, one male rESC line was also included in our analysis. As in mouse, we found that *Xist* upregulation occurs exclusively in female rat cells between day 2 and day 4 of differentiation (Fig. 2A). In contrast, *Tsix* pinpoint signals decrease upon differentiation in all differentiating rESC lines that we tested (Fig. 2A, and Supplementary Fig. 2). Next, we addressed the dynamics of *Xist* expression by performing *Xist* RNA FISH analysis at different time points upon neuronal differentiation. In undifferentiated rESCs, *Xist* RNA pinpoint signals were observed within the nuclei of both female and male cells (Fig. 2B). However, since the *Xist* RNA FISH probe can hybridize to both *Xist* and *Tsix* RNA, the pinpoint signal might represent *Tsix* expression instead of *Xist*. Around day 2 of neuronal differentiation, *Xist* RNA starts to accumulate exclusively on a single X chromosome within female nuclei, whereas *Xist* RNA accumulation was never observed in differentiating male rESCs (Fig. 2B, lower panel). Importantly, upon differentiation of A10p20 and A4p20 rESC female lines, more than 60% of the nuclei showed an *Xist* RNA-coated X chromosome at day 6 of differentiation (Fig. 2C). Taken together, these observations show that neuronal differentiation of rESCs in absence of 2i inhibitors allows *Xist* RNA to be upregulated and spread *in cis* from a single X chromosome in female cells.

**Figure 3:**
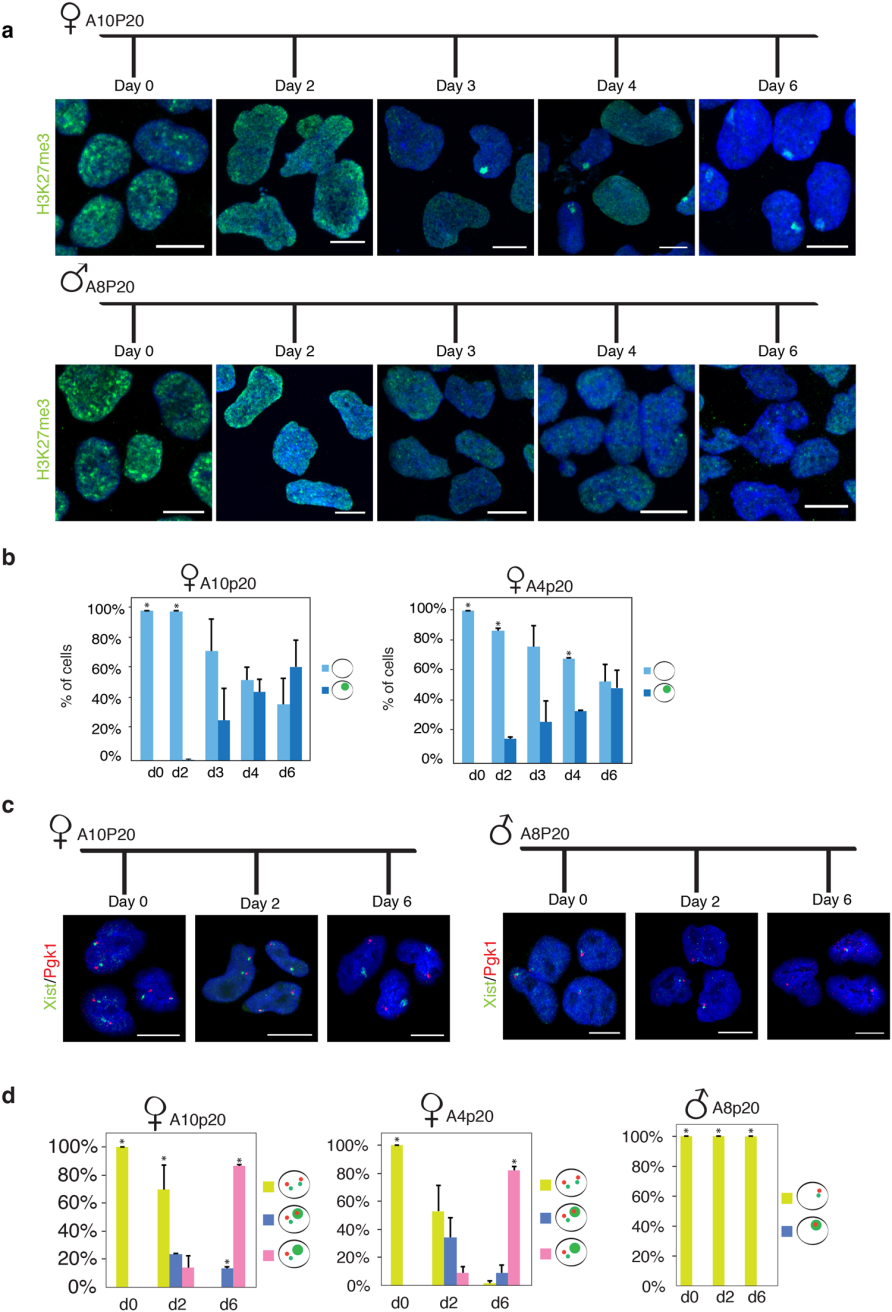
*Xist*-mediated silencing of X-linked genes. (**a**) Representative images of H3K27me3 (green) immunofluorescence analysis in female (A10p20–upper panel) and male (A8p20-lower panel) rESC at different time points upon neuronal differentiation. DNA is stained with DAPI (blue). Scale bars represent 10 μm. (**b**) Quantification of relative number of cells carrying a H3K27me3 domain at day 0, 2, 3, 4 and 6 of neuronal differentiation. Data of A10p20 (left graph) and A4p20 (right graph) female rESC lines are shown. H3K27me3 domains were never noticed in male nuclei. More than 100 nuclei were counted for each time point and in each experimental replicate. Error bars represent standard deviations between two experiments. Asterisks indicate significant differences between the two groups (absence versus presence of domain) within each differentiation time point. The p-values are the given: p-value_A10-d0_ < 0.0001, p-value_A10-d2_ = 0.003 and p-value_A4-d0_ < 0.0001, p-value_A4-d2_ = 0.03, p-value_A4-d4_ = 0.04. (**c**) Representative images of *Xist* (green)/*Pgk1* (red) two-colour RNA-FISH in both female (A10p20-upper panel) and male (A8p20-lower panel) D0 and D6 differentiating rat cells are shown. DNA is stained with DAPI (blue). Scale bars represent 10 μm. (**d**) *Xist* (green)/*Pgk1* (red) two-colour RNA-FISH quantitative analysis at different time points during neuronal differentiation of female (A10p20-upper panel) and male (A8p20-lower panel) rESCs. The relative number of cells showing either biallelic or monoallelic *Pgk1* expression is quantified, together with the relative number of cells carrying *Xist* pinpoints or cloud signals. More than 100 nuclei were counted for each time point and in each experiment, with exception of A10p20 experimental replicate 2, where at day 2, 50 nuclei were counted. Error bars represent standard deviation of two experimental replicates. Asterisks indicate significant differences between groups (biallelic expression versus biallelic expression and *Xist* cloud versus monoallelic expression) within each differentiation time point. P-values are provided: p-value_A10-d0bial. VS A10-d0bial.&cloud_ < 0.0001, p-value_A10-d0bial. VS A10-d0monal._ < 0.0001, p-value_A10-d2bial. VS A10-d2monal._ = 0.049, p-value_A10-d6bial. VS A10-d6monal._ < 0.0001, p-value_A10-d6bial.&cloud VS A10-d6monal._ < 0.0001, p-value_A10-d6bial. VS A10-d6bial&cloud_. = 0.00076 and p-value_A4-d0bial. VS A4-d0bial.&cloud_ < 0.0001, p-value_A4-d0bial. VS A4-d0monal._ < 0.0001, p-value_A4-d6bial. VS A4-d6monal._ = 0.0047, p-value_A4-d6bial.& VS A4-d6bial.&cloud_ = 0.003 and p-value_A8-d0monal. VS A8-d0monal.&cloud_ < 0.0001 p-value_A8-d2monal. VS A8-d2monal.&cloud_ < 0.0001, p-value_A8-d6monal. VS A8-d6monal.&cloud_ < 0.0001.

In mouse, the H3K27me3 histone modification associated with gene silencing represents one of the earliest histone modifications that accumulates on the Xi during XCI^77–79^. Therefore, we monitored enrichment of H3K27me3 by immunofluorescence analysis upon differentiation of both male and female rESCs. In undifferentiated rESCs, no H3K27me3 domains were observed in neither male nor female cells (Fig. 3A). However, starting from day 2 of differentiation and in line with female-specific upregulation of *Xist* RNA, H3K27me3 started to accumulate into specific nuclear domains within female cells. By day 6, more than 60% of the female nuclei showed one H3K27me3 domain, thus confirming that XCI is efficiently initiated upon female rESCs differentiation (Fig. 3B). Finally, to precisely assess the dynamics of X-linked gene silencing, we followed the *Xist*-mediated inactivation of the X-linked genes *Pgk1* and *Rnf12* by two-colour RNA-FISH analysis at different time points upon rESCs differentiation. While the single copy of *Pgk1* and *Rnf12* in male cells remains actively transcribed throughout differentiation, the transcriptional inactivation of one copy of both X-linked genes in female cells starts around day 2 of differentiation (Fig. 3C and Supplementary Fig. 3). At day 6 of differentiation inactivation of X-linked genes is reached in up to 70% of the female nuclei (Fig. 3C and Supplementary Fig. 3).

### Overexpression of RNF12 leads to *Xist* activation

**Figure 4:**
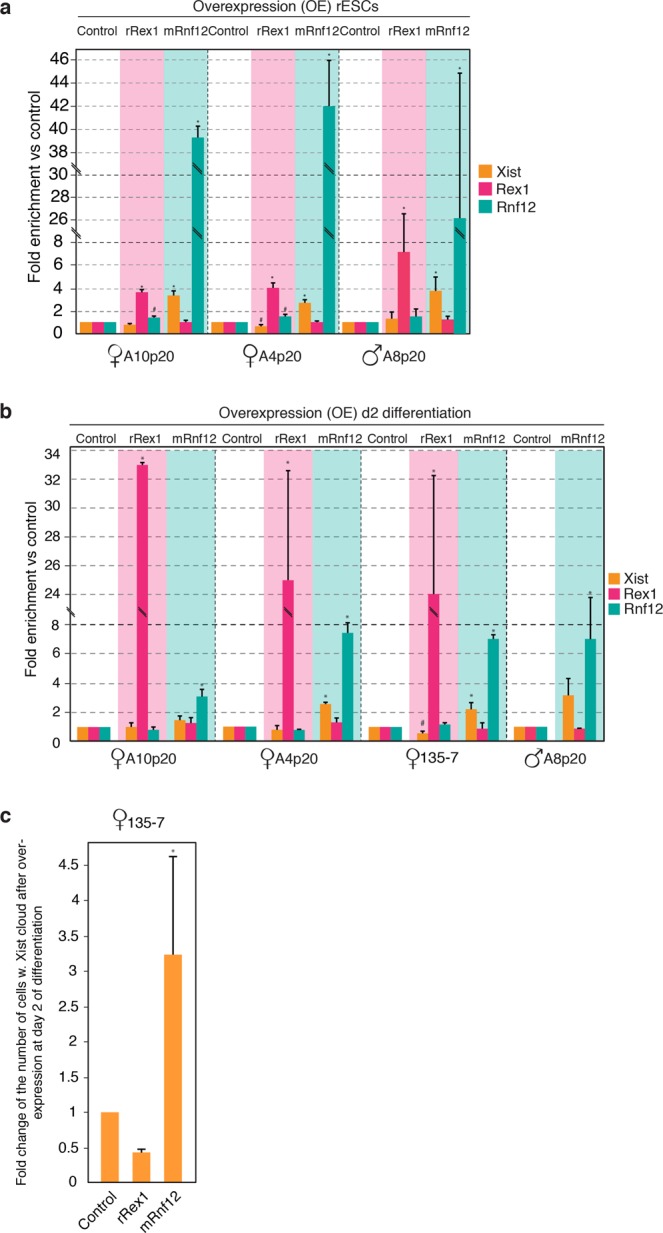
RNF12 and REX1 overexpression in rESCs and differentiating rESCs. (**a**) qPCR analysis of *Xist*, *Rex1* and *Rnf12* expression levels after overexpression of rat REX1 and mouse RNF12 proteins in undifferentiated rat ESCs. The controls (overexpression of empty vectors) were set to 1 for every gene analysed in each cell line used. Error bars represent standard deviation between technical replicates of three experiments. Asterisks and hashtags indicate significant differences, with the following p-values: p-value_A10_Xist-Control VS *Xist*-OE_Rnf12_ = 0.02, p-value_A10_Xist-OE_Rex1 VS *Xist*-OE_Rnf12_ = 0.03, p-value_A10_Rnf12-Control VS Rnf12-OE_Rnf12_ < 0.001, p-value_A10_Rnf12-OE_Rex1 VS Rnf12-OE_Rnf12_ < 0.001, p-value_A10_Rnf12-OE_Rnf12 VS Rnf12-OE_Rex1_ < 0.001, p-value_A10_Rex1-Control VS Rex1-OE_Rex1_ < 0.001, p-value_A10_Rex1-OE_Rex1 VS Rex1-OE_Rnf12_ < 0.001, (#)p-value_A10_Rnf12-Control VS Rnf12-OE_Rex1_ = 0.01, p-value_A4_Xist-Control VS *Xist*-OE_Rnf12_ < 0.001, p-value_A4_Xist-OE_Rex1 VS *Xist*-OE_Rnf12_ = 0.001, (#)p-value_A4_Xist-Control VS *Xist*-OE_Rex1_ = 0,002, p-value_A4_Rnf12-Control VS Rnf12-OE_Rnf12_ < 0.001, p-value_A4_Rnf12-OE_Rex1 VS Rnf12-OE_Rnf12_ < 0.001, (#)p-value_A4_Rnf12-Control VS Rnf12-OE_Rex1_ = 0.06, p-value_A4_Rex1-Control VS Rex1-OE_Rex1_ < 0.001, p-value_A4_Rex1-OE_Rex1 VS Rex1-OE_Rnf12_ < 0.001, p-value_A8_Xist-Control VS *Xist*-OE_Rnf12_ = 0.02, p-value_A8_Xist-OE_Rex1 VS *Xist*-OE_Rnf12_ = 0.03, p-value_A8_Rnf12-Control VS Rnf12-OE_Rnf12_ = 0.02, p-value_A8_Rnf12-OE_Rex1 VS Rnf12-OE_Rnf12_ = 0.02, p-value_A8_Rex1-Control VS Rex1-OE_Rex1_ = 0.02, p-value_A8_Rex1-OE_Rex1 VS Rex1-OE_Rnf12_ = 0.02. (**b**) qPCR analysis of *Xist*, *Rex1* and *Rnf12* expression levels after overexpression of rat REX1 and mouse RNF12 proteins in day 2 differentiating rat ESCs to neurons. The controls (overexpression of empty vectors) were set to 1 for every gene analysed in each cell line used. Error bars represent standard deviation between technical replicates from two experiments. Asterisks indicate significant differences with the following p-values: p-value_A10_Rnf12-Control VS Rnf12-OE_Rnf12_ = 0.007, p-value_A10_Rnf12-OE_Rex1 VS Rnf12-OE_Rnf12_ = 0.02, p-value_A10_Rex1-Control VS Rex1-OE_Rex1_ < 0.001, p-value_A10_Rex1-OE_Rex1 VS Rex1-OE_Rnf12_ < 0.001, p-value_A4_Xist-Control VS *Xist*-OE_Rnf12_ < 0.001, p-value_A4_Xist-OE_Rex1 VS *Xist*-OE_Rnf12_ < 0.001, p-value_A4_Rnf12-Control VS Rnf12-OE_Rnf12_ = 0.02, p-value_A4_Rnf12-OE_Rex1 VS Rnf12-OE_Rnf12_ < 0.001, p-value_A4_Rex1-Control VS Rex1-OE_Rex1_ < 0.001, p-value_A4_Rex1-OE_Rex1 VS Rex1-OE_Rnf12_ < 0.001, p-value_135-7_Xist-Control VS *Xist*-OE_Rnf12_ < 0.01, p-value_135-7_Xist-OE_Rex1 VS *Xist*-OE_Rnf12_ < 0.001, (#)p-value_135-7_Xist-Control VS *Xist*-OE_Rex1_ = 0.007, p-value_135-7_Rnf12-Control VS Rnf12-OE_Rnf12_ < 0.001, p-value_135-7_Rnf12-OE_Rex1 VS Rnf12-OE_Rnf12_ < 0.001, p-value_135-7_Rex1-Control VS Rex1-OE_Rex1_ < 0.001, p-value_135-7_Rex1-OE_Rex1 VS Rex1-OE_Rnf12_ < 0.001, p-value_A8_Rnf12-Control VS Rnf12-OE_Rnf12_ = 0.03. (**c**) Fold quantification of number of cells with *Xist* RNA clouds in 135-7 female line after overexpression of empty vector (control, set to 1), *rRex1* and *mRnf12* in rESCs at day 2 after initiation of differentiation towards neurons. Error bars represent standard variation between technical replicates from two experiments. The asterisks indicates significant difference with the following p-values: p-value_Control VS mRnf12_ = 0,002 and p-value_rRex1 VS mRnf12_ = 0,01.

The X-linked E3 ubiquitin ligase RNF12 has been previously shown to activate *Xist* transcription at the onset of XCI^25,27^. Importantly, the pluripotency factor REX1 has been identified as a key target of RFN12, and dose-dependent degradation of REX1 by RNF12 has been proposed to act as a crucial mechanism directing initiation of XCI upon differentiation of female mESCs^26^. Since the RNF12-REX1 axis represents an important pathway for XCI initiation in mouse, we asked whether these factors play similar roles in rat XCI. To this end, we transiently overexpressed *Rnf12* and *Rex1* in rESCs, and determined the impact of overexpression on *Xist* RNA regulation. Based on findings in mouse, we expected REX1 overexpression to result in the inhibition of *Xist* transcription whereas overexpressing RNF12 would lead to *Xist* up-regulation^26,27,80^. Since the catalytic ring finger domain of RNF12 shows 100% of amino acid sequence identity between mouse and rat, and human RNF12 transgenes induce ectopic XCI in mouse ESCs^25^, we overexpressed the mouse RNF12 protein (mRNF12) in rESCs. Contrarily, as the zinc finger domain of REX1 is less conserved between the two species, overexpression of REX1 was achieved by transfecting rESCs with rat REX1 cDNA (rREX1). *Xist* RNA expression levels were determined by qPCR analysis, and the experiment was performed in three independent undifferentiated rESC lines as well as upon neuronal differentiation (Fig. 4A,B). Overexpression of mRNF12 consistently resulted in upregulation of *Xist* RNA in both male and female rESCs prior to and during rESCs differentiation, thus indicating RNF12 to act as important trans-acting activator of *Xist* in rat (Fig. 4A,B). *Xist* RNA FISH analysis performed at day 2 of neuronal differentiation upon mRNF12 overexpression further confirmed the impact of RNF12 on XCI initiation (Fig. 4C). However, the impact of rREX1 overexpression on *Xist* regulation, prior to and upon differentiation of rESCs, appeared less consistent (Fig. 4A–C). Although RNA-FISH and qPCR analysis indicated a decrease in *Xist* expression when rREX1 is overexpressed for one rESC line at day 2 of neuronal differentiation (line 135-7), *Xist* down-regulation was not significant for most comparisons. These results may suggest that *Rex1* plays a less prominent role in XCI in rat, but could also be explained by our experimental setup, as previously observed in mouse ESCs^26^, where upregulation of *Xist* mediated by *Rnf12* over-expression was also easier to detect than *Rex1* over-expression mediated down-regulation of *Xist*. Although the role of *Rex1* in XCI in rat needs further investigation, our findings indicate a role for *Rnf12* in XCI in rat, providing a powerful new model system to elucidate the complex mechanisms directing initiation of XCI.

## Discussion

Our knowledge concerning the regulation of XCI in developing rat embryos is limited and relies on conservation of the key regulators *Xist* and *Tsix* between mouse and rat and a few studies in which, similar to mouse, iXCI has been proposed to occur in early rat embryonic development^30,37–40^. Studying the XCI process in rESCs offers the opportunity to explore species-specific epigenetic features and will help to reach a more comprehensive understanding of the XCI process in mammals. Although rESCs *in vitro* differentiation protocols have been previously established^72,74,75^, XCI studies were limited, only examining *Xist* expression and accumulation of chromatin modifications. Here, we confirm the accumulation of XCI-related epigenetic features in rESCs during differentiation according to the previously established protocols, and further show that this is accompanied by proper down-regulation of pluripotency markers^81^. We also showed that transcriptional inactivation of X-linked genes directly follows *Xist* RNA accumulation on one of the two X chromosomes. In fact, the exclusive enrichment of H3K27me3 loci in female nuclei starts around day 3 of neuronal differentiation, and *Xist*-mediated silencing of *Pgk1* and *Rnf12* occurs with similar dynamics.

Overexpression of mRNF12 protein in rESCs efficiently recapitulates RNF12 function in mouse as an activator of *Xist* expression. RNF12 is highly conserved among mammals^82^. The observed up-regulation of rat *Xist* upon mRNF12 overexpression is in line with our previous findings that overexpression of human RNF12 leads to ectopic *Xist* expression and XCI in mESCs^25^, indicating that the role of RNF12 in XCI is highly conserved in mammals. In contrast, rREX1 over-expression does not lead to consistent down-regulation of *Xist* levels as was expected based on our mouse studies. This result may indicate the presence of an alternative pathway by which RNF12 activates XCI in rat, a theory that is supported by the lower level of conservation in the REX1 zinc finger domain. On the contrary, REX1 represents the only target of RNF12 in mouse ESCs identified so far. In addition, measuring down-regulation of *Xist* expression in the present setting after transient transfection is technically more challenging than detecting up-regulation of *Xist*, and may require more sensitive approaches to experimentally define the exact role of *Rex1* in XCI in rat. Additional alternative approaches, such as inducible over-expression of *Rex1*, and ChIP sequencing studies should shed more light on the exact role of *Rex1* in XCI regulation.

In conclusion, we were able to set up a robust *in vitro* system to study the regulation of XCI in differentiating rESCs and our results suggest that the main steps of XCI in our rat *in vitro* system are highly similar to those of mouse XCI. The generation of hybrid F1 polymorphic rESCs together with the application of the CRISPR/Cas9 technology for genomic editing to the rat system will increase the use of rat as a model organism in basic epigenetic and biomedical research.

## Methods

### Cell culture and DNA transfection

rESCs were derived as previously described^56^ and subsequently maintained in N2B27 medium supplemented with 3 μM CHIR99021 (Stemgent), 1 μM PD0325901 and 1000 U/ml mouse LIF on mouse feeders.

For monolayer differentiation culture plates were coated with 100 μg/ml laminin (Sigma-Aldrich) for at least 4 hours at 37 °C, followed by three PBS washes. Single rESCs were plated at a density of 10^5^/cm^2^ for the female cell lines and 2 × 10^4^/cm^2^ for the male cell lines in N2B27 supplemented with 10 μM of ROCK inhibitor (Sigma-Aldrich) for the first three days. Thereafter, the ROCK inhibitor was eliminated. Medium was refreshed daily.

For overexpression experiments, the m*Rex1*, r*Rex1* and m*Rnf12* coding sequences were subcloned into pCAG-Flag, a CAG-driven expression vector containing a Flag-tag. RESCs were transfected using lipofectamine 2000 (Invitrogen) according to the manufacturer’s instructions, followed by 48 hours of puromycin selection (1,5 μg/ml). Overexpression in differentiating cells was performed as follows: rESCs were trypsinised and plated at a density of 1,3*10^5^/cm^2^ in gelatinized 6-well plates in 2i media supplemented with 10 μM of ROCK inhibitor (Sigma-Aldrich) without feeders. The next day cells were transfected using lipofectamine 2000 (Invitrogen) according to the manufacturer’s instructions. Cells were left to recover in 2i media overnight and then a 48 hour-puromycin selection (0,25 μg/ml for the A10p20, A4p20 and A8p20 cell lines and 1 μg/ml for the 135-7 cell line) was initiated in N2B27 differentiation media.

### Probe preparation and Fluorescent *in Situ* Hybridization (FISH)

For preparing probes detecting *Xist*, *Pgk1* and *Rnf12* mRNAs, BACs harboring these genes were labelled as a whole, with digoxigenin and biotin (Roche) respectively, by nick translation following the manufacturer’s instructions.

For RNA-FISH at different time points of neuronal differentiation and after overexpression experiments during differentiation, cells were grown on glass coverslips and then fixed with 3% PFA for 10 minutes on ice, followed by three washes in PBS. Next, cells were permeabilised with 0.5% Triton and washed again three times in PBS. Cytoplasm was removed by treating the cells with 0.025% pepsin in 0.01 N HCL for 3 minutes at 37 °C. Subsequently, cells were dehydrated with sequential ethanol washes (70%, 85% and 100% 2 minutes each) and air-dried. Finally, probes were applied on the samples overnight at 37 °C in a 50% Formamide/2XSSC humid chamber. The next day, slides were washed two times, 5 minutes each, in 50% Formamide/2xSSC pH = 7.4 at 37 °C, followed by two washes, 5 minutes each in 2xSSC at 37 °C and cells were blocked for 30 minutes at room temperature with TSBSA (2 mg/ml bovine serum albumin in 0.1 M Tris and 0.15 M NaCl) in a humid chamber at room temperature. Detection was performed by incubation with anti-digoxigenin FITC (Boehringer, 1:250) and streptavidin alexa fluor 555 (Thermofisher Scientific, 1:400) in TSBSA for 30 minutes at room temperature. Slides were then washed two times, 5 minutes each with TS (0.1 M Tris, 0.15 M NaCl) and mounted with ProLong® Gold Antifade Mountant with Dapi (ThermoFisher Scientific). Imaging was performed on a Zeiss LSM700 microscope (Carl Zeiss, Jena).

### Expression analysis

Cells were lysed by direct addition of 500 μl of TRIZOL and total RNA was extracted according to the manufacturer’s instructions (Invitrogen). To remove genomic DNA contamination, samples were treated 15 minutes at 37 °C with DNaseI (Invitrogen). Next, 1 μg of RNA was reverse transcribed by Superscript II reverse transcriptase with random hexamers (Invitrogen). For quantitative PCR (qPCR) gene expression levels were quantified using 2x SYBR Green PCR Master Mix (Applied Biosystems) in a CFX384 Real-Time machine (Bio-Rad). Expression levels were normalized to *Actinb* using the Δ-CT method. The primers used were as follows: *Xist* forward TGCCTGGATTTAGAGGAG and reverse CTCCACCTAGGGATCGTCAA, *Tsix* forward CGGACTATCTCCGCTTCTTG and reverse CACCTCTCTGGCTTCTTCCA, *Nanog* forward TAGCCCTGATTCTTCTAGCA and reverse TTTGCTGCAACGGCACATAA, *Essrb* forward GGCGTTCTTCAAGAGAACCA and reverse CCCACTTTGAGGCATTTCAT, *Prdm14* forward AGGAACTGCGCTTCGTTCT and reverse GGCATCACCAAAAGCTGTCT, *Rex1* forward AAATCATGACGAGGCAAGGC and reverse TGAGTTCGCTCCAACAGTCT, *Nestin* forward CTCTGCTGGAGGCTGAGAAC and reverse TGGTATCCCAAGGAAATTCG, *Actinb* forward GCTGGCCTTAGAGACCACAG and reverse AAGCAATTCAGCAACACCAA.

### Immunocytochemistry

For immunofluorescence analysis of different time points of neuronal differentiation and of overexpression experiments, cells were grown on glass coverslips and then fixed with 3% PFA for 10 minutes at room temperature followed by three washes in PBS (3 × 5′). Thereafter, cells were permeabilised with 0.5% Triton, washed with PBS (3 × 5′) and blocked with 2% BSA, 5% donkey serum in PBS (blocking solution) for 30 minutes at room temperature. This was followed by anti-H3K27me3 rabbit (Diagenode, 1:500) incubation, diluted in blocking solution, at 4 °C overnight in a humid chamber. The next day, slides were washed in PBS (3 × 5′) and blocked with donkey anti-rabbit alexa fluor 488 (ThermoFischer Scientific, 1:250) secondary antibody, diluted in blocking solution for 1 hour at room temperature in a humid chamber. Slides were then washed in PBS (3 × 5′) and mounted with ProLong® Gold Antifade Mountant with Dapi (ThermoFisher Scientific). Confocal imaging was performed on a Zeiss LSM700 microscope (Carl Zeiss, Jena).

### Statistical analysis

Statistical significance between the different groups was assessed by student t-test. Statistical significance was set at p < 0.05.

## Supplementary information

Supplemental Information
