# Supplementary material for: A novel approach to differentiate rat embryonic stem cells in vitro reveals a role for RNF12 in activation of X chromosome inactivation
Source: Sci Rep. 2019 Apr 15;9:6068. doi: 10.1038/s41598-019-42246-2 (PMC6465393; doi:10.1038/s41598-019-42246-2)
Supplement: Supplementary file 1 — Supplemental Information [file 41598_2019_42246_MOESM1_ESM.pdf]

Supplemental Information to:

A novel approach to differentiate rat embryonic stem cells *in vitro* reveals a role for RNF12 in activation of X chromosome inactivation

Aristea Magaraki[1], Agnese Loda[1,2], Cristina Gontan[1], Sarra Merzouk[1], Esther Sleddens-Linkels[1], Stephen Meek[2], Willy M. Baarends[1], Tom Burdon[2] and Joost Gribnau[1*]

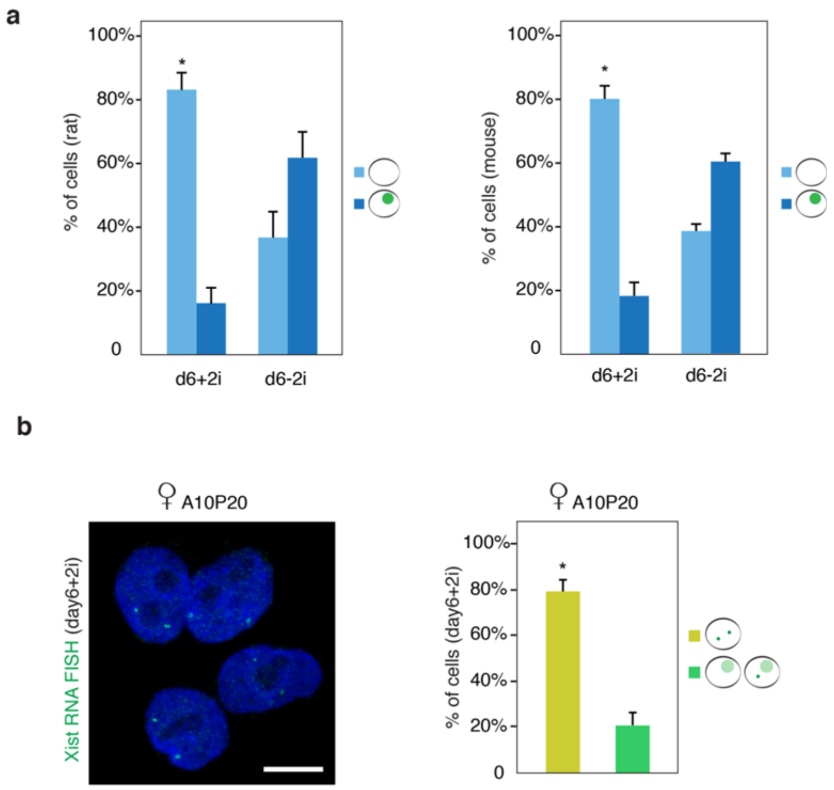

Sup. Figure 1. Delayed XCI in the presence of MEK and GSK3β inhibitors.

(**a**) Quantification of H3K27me3 immunofluorescence analysis in female clone A10p20 (left) and in mouse ES cells (F12 strain, right) at day 6 of neural differentiation in the presence (+2i) or absence (-2i) of MEK and GSK3β inhibitors. Error bars indicate standard deviation of two experiments. Asterisks indicate significant differences with the following p-values: p-value (rat_d6+2i) $_{\text{cloud VS no cloud}}$=0.008 and p-value (mouse_d6+2i) $_{\text{cloud VS no cloud}}$=0.006.

(**b**) Left: Representative image of *Xist* RNA FISH analysis performed at day 6 of neuronal differentiation of female clone A10P20 in the presence of MEK and GSK3β inhibitors. *Xist* is shown in FITC (green) and DNA is stained with DAPI (blue). Scale bar represents 10 μm. Right: Quantification of cells showing pinpoints or clouds at day 6 of neuronal differentiation in the presence of MEK and GSK3β inhibitors. Error bars represent standard deviation between two experiments. Asterisk shows significant difference with p-value$_{\text{pinpoints VS clouds}}$=0.008.

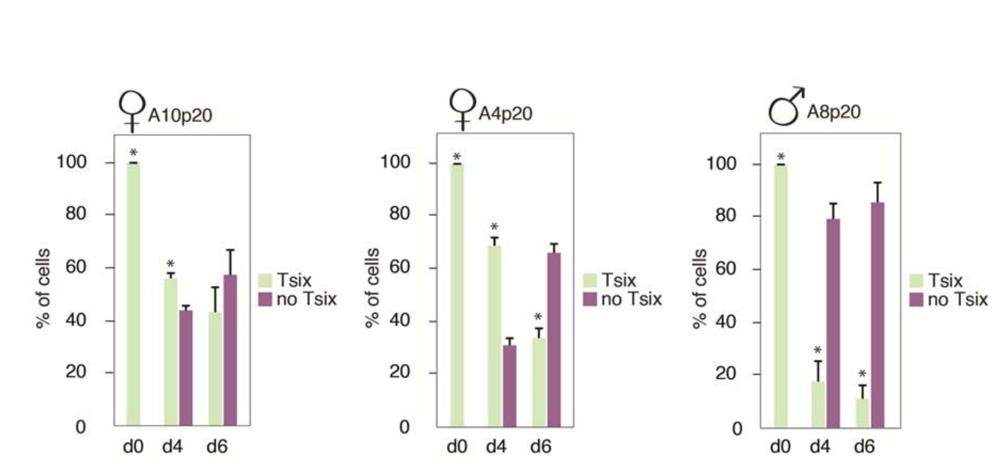

Sup. Figure 2. Quantification of Tsix pinpoints during neuronal differentiation. Quantification of relative number of cells with *Tsix* RNA signals (pinpoint) and without (no pinpoint) in A10p20 (left graph), A4p20 (middle graph) female and A8P20 (right graph) male rESCs at day 0, 4 and 6 neuronal differentiation. More than 100 nuclei were counted for each time point and for each experiment. Error bars indicate standard deviation between two experiments. Asterisks indicate significant difference between the two groups (signal (Tsix) versus no signal (no Tsix)) within each differentiation time point. P-values are provided: p-value$_{\text{A10-d0 Tsix VS no Tsix}}$<0.001, p-value$_{\text{A10-d2 Tsix VS no Tsix}}$=0.037, p-value$_{\text{A4-d0 Tsix VS no Tsix}}$<0.001, p-value$_{\text{A10-d2 Tsix VS no Tsix}}$=0.006, p-value$_{\text{A10-d6 Tsix VS no Tsix}}$=0.01, p-value$_{\text{A8-d0 Tsix VS no Tsix}}$<0.001, p-value$_{\text{A8-d2 Tsix VS no Tsix}}$=0.009, p-value$_{\text{A8-d6 Tsix VS no Tsix}}$=0.01.

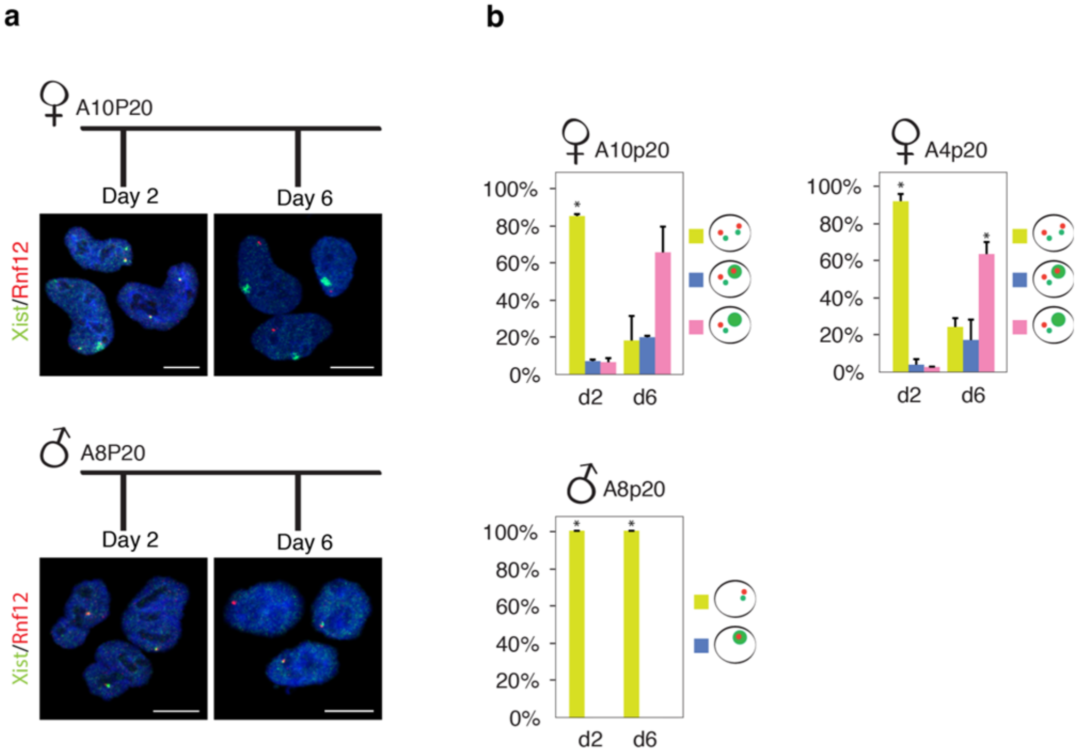

Sup. Figure 3. ***Xist*****-mediated silencing of the X-linked gene** ***Rnf12*****.**

(a) Representative images of *Xist* (green)/*Rnf12* (red) two-colour RNA-FISH in both female (A10p20-upper panel) and male (A8p20-lower panel) D2 and D6 differentiating rat cells are shown. DNA is stained with DAPI (blue). Scale bars represent 10 µm. (**b**) *Xist* (green)/*Rnf12* (red) two-colour RNA-FISH quantitative analysis at day2 (d2) and day 6 (d6) time points upon neuronal differentiation of female (A10p20-upper panel) and male (A8p20-lower panel) rESCs. The relative number of cells showing either biallelic or monoallelic *Rnf12* expression is quantified, together with the relative number of cells carrying *Xist* pinpoints or cloud signals. More than 100 nuclei were counted for each time point and in each experiment. Asterisks indicate significant differences between groups (*Rnf12* biallelic expression versus biallelic expression and *Xist* cloud versus *Rnf12* monoallelic expression) within each differentiation time point. P-values are: p-value$_{A10\text{-}d2bial\ VS\ A10\text{-}d2bial\&cloud}<0.001$, p-value$_{A10\text{-}d2bial\ VS\ A10\text{-}d2monal}<0.002$, p-value$_{A10\text{-}d6bial\&cloud\ VS\ A10\text{-}d6monal}=0.004$ and p-value$_{A4\text{-}d2bial\ VS\ A4\text{-}d2bial\&cloud}=0.001$, p-value$_{A4\text{-}d2bial\ VS\ A4\text{-}d2monal}=0.004$, p-value$_{A4\text{-}d6bial\ VS\ A4\text{-}d6monoal}=0.04$, p-value$_{A4\text{-}d6bial\&cloud\ VS\ A4\text{-}d6monal}=0.02$ and p-value$_{A8\text{-}d2monoal.\ VS\ A8\text{-}d2monol.\&cloud}<0.001$, p-value$_{A8\text{-}d6monoal.\ VS\ A8\text{-}d6monol.\&cloud}<0.0001$.
